# Endoscopic Transforaminal Thoracic Foraminotomy and Discectomy for the Treatment of Thoracic Disc Herniation

**DOI:** 10.1155/2013/264105

**Published:** 2013-12-18

**Authors:** Hong-Fei Nie, Kai-Xuan Liu

**Affiliations:** ^1^Department of Orthopaedic Surgery, West China Hospital, Sichuan University, Chengdu, Sichuan 610041, China; ^2^Atlantic Spine Center, 475 Prospect Avenue, Suite 110, West Orange, NJ 07052, USA

## Abstract

Thoracic disc herniation is a relatively rare yet challenging-to-diagnose condition. Currently there is no universally accepted optimal surgical treatment for symptomatic thoracic disc herniation. Previously reported surgical approaches are often associated with high complication rates. Here we describe our minimally invasive technique of removing thoracic disc herniation, and report the primary results of a series of cases. Between January 2009 and March 2012, 13 patients with symptomatic thoracic disc herniation were treated with endoscopic thoracic foraminotomy and discectomy under local anesthesia. A bone shaver was used to undercut the facet and rib head for foraminotomy. Discectomy was achieved by using grasper, radiofrequency, and the Holmium-YAG laser. We analyzed the clinical outcomes of the patients using the visual analogue scale (VAS), MacNab classification, and Oswestry disability index (ODI). At the final follow up (mean: 17 months; range: 6–41 months), patient self-reported satisfactory rate was 76.9%. The mean VAS for mid back pain was improved from 9.1 to 4.2, and the mean ODI was improved from 61.0 to 43.8. One complication of postoperative spinal headache occurred during the surgery and the patient was successfully treated with epidural blood patch. No other complications were observed or reported during and after the surgery.

## 1. Introduction

Thoracic disc herniation is an uncommon condition. Although conservative treatment works well for many patients with thoracic disc herniation, surgical treatment is needed for patients suffering from myelopathy and/or neurological deficit caused by thoracic disc herniation. In the past decade, quite a few surgical procedures have been reported in the literature, and each of them has its own advantages and disadvantages [1–14]. Currently there is no universally accepted optimal surgical treatment for symptomatic thoracic disc herniation.

Minimally invasive spine surgery has proven safe and effective in treating lumbar and cervical herniations [15–24]. The advantages of minimally invasive techniques have compelled many physicians to explore the feasibility of using minimally invasive techniques in treating thoracic disc herniation, and a number of authors have reported encouraging primary results [14, 25–28]. Based on our extensive experience with treating lumbar and cervical disc herniation using minimally invasive techniques, we have developed an endoscopic transforaminal foraminotomy and discectomy technique for treating thoracic disc herniation. The purposes of this paper are to describe the technique and to report the results of a series of cases.

## 2. Materials and Methods

Between January 2009 and January 2012, 13 patients with symptomatic thoracic disc herniation were treated with percutaneous endoscopic thoracic foraminotomy and discectomy. The surgical procedures were performed under local anesthesia at our outpatient surgical center. All patients had soft thoracic disc herniation confirmed with magnetic resonance imaging (MRI). Symptoms related to the herniation were confirmed using discography. After a mean of 17 months of followup (range: 6–41 months), we analyzed the clinical outcomes using the visual analogue scale (VAS), MacNab classification, and Oswestry disability index (ODI).

### 2.1. Diagnosis and Patient Selection

Considering that patients with thoracic disc herniation may have varied symptoms, some of which may be similar to symptoms of other medical conditions, we made the diagnosis by reviewing the patients' medical history, performing physical examination, and analyzing radiographic findings. Patients qualified for our surgical procedure met the following criteria. First, the patient had middle back pain with or without radiation. Second, conservative pain treatments had failed to alleviate the pain. Third, magnetic resonance imaging (MRI) revealed soft thoracic disc herniation. And finally discography confirmed painful disc before the surgical procedure.

Patients with calcified discs or hard disc herniations were not treated with this procedure.

### 2.2. Tools

During the surgical procedure, a burr, a bone shaver, and the Holmium-YAG laser were used to undercut the facet and rib head for foraminotomy. Discectomy was achieved by using a grasper, radiofrequency, and the Holmium-YAG laser. The surgical procedures were performed with the assistance of an 8 mm (outer diameter) Wolf endoscope (Richard Wolf Medical Instruments Corporation, Vernon Hills, IL, USA).

### 2.3. Surgical Technique

**Figure 1:**
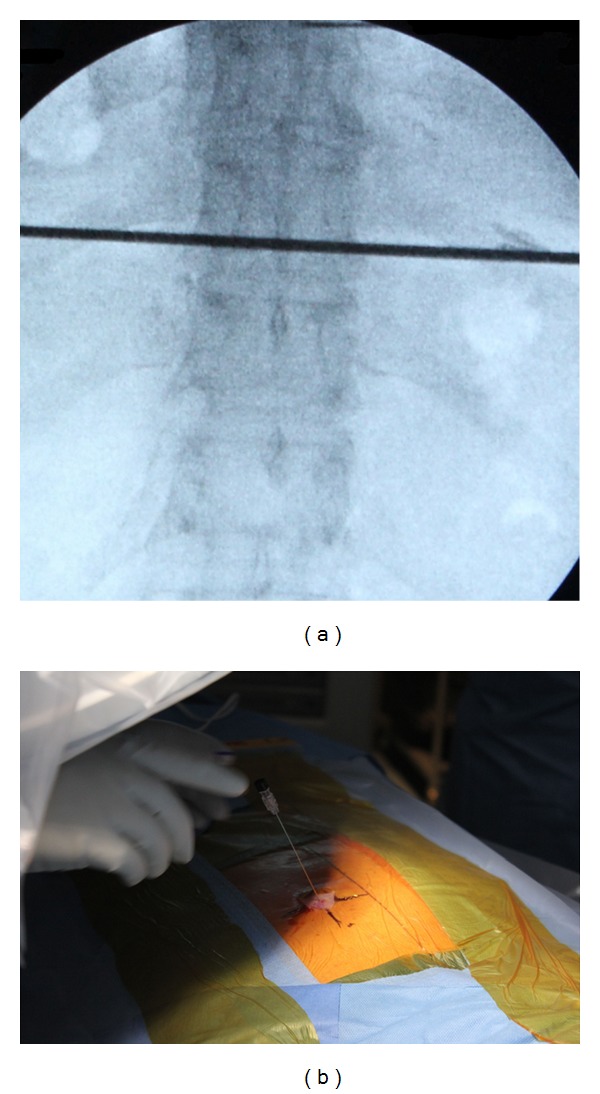
The target disc was identified under fluoroscopic guidance (a), and the entry point between the rib head and the facet was marked on the skin (b).

**Figure 2:**
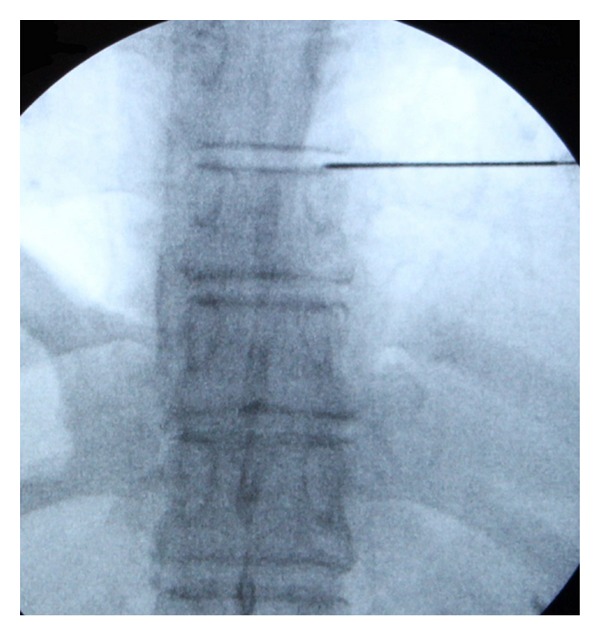
Discography was performed to confirm the target disc and to help identify the location of the herniation; the needle was parallel to the upper endplate of the lower vertebral body.

The procedures were performed under local anesthesia with the patient in a prone position on a radiolucent table. The target disc was identified under fluoroscopic guidance (Figure 1(a)), and the entry point between the rib head and the facet (on oblique view) was marked on the skin (Figure 1(b)). Discography was performed to confirm the target disc and to help identify the location of the herniation. The 18 G needle inserted to perform discography was parallel to the upper endplate of the lower vertebral body (Figure 2). The tip of the needle reached posterior disc margin (on the lateral view) and was situated between midline and medial pedicle line (on the AP view). The surgical region was anesthetized with a combination of 0.5% lidocaine and epinephrine.

**Figure 3:**
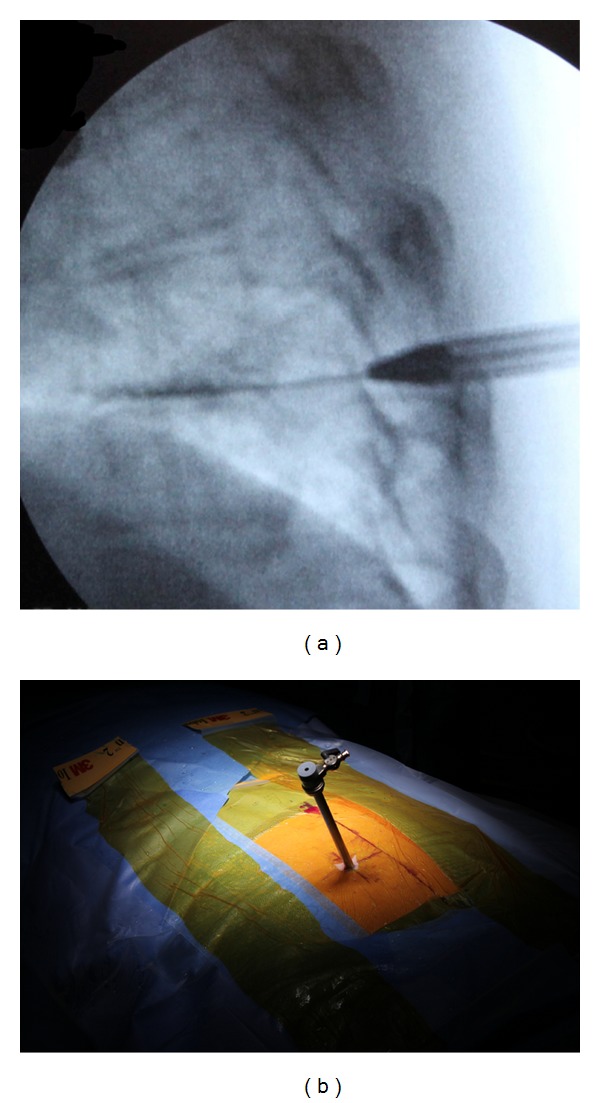
A sequential dilator was then inserted over the wire towards the posterolateral margin of the facet (a). A working cannula was guided to the extraforaminal region over the dilator (b).

After discography, a guiding wire was inserted through the needle, and a 10 mm skin incision was subsequently made. The needle was removed, and a sequential dilator was then inserted over the wire towards the posterolateral margin of the facet (Figure 3(a)). Once the tip of the dilator reached the surface of the annulus, the guiding wire was removed and the dilator was further inserted into the target foramen. A working cannula was then guided to the extraforaminal region over the dilator (Figure 3(b)). At this juncture, the dilator was removed and the endoscope was placed to assist with visualization.

**Figure 4:**
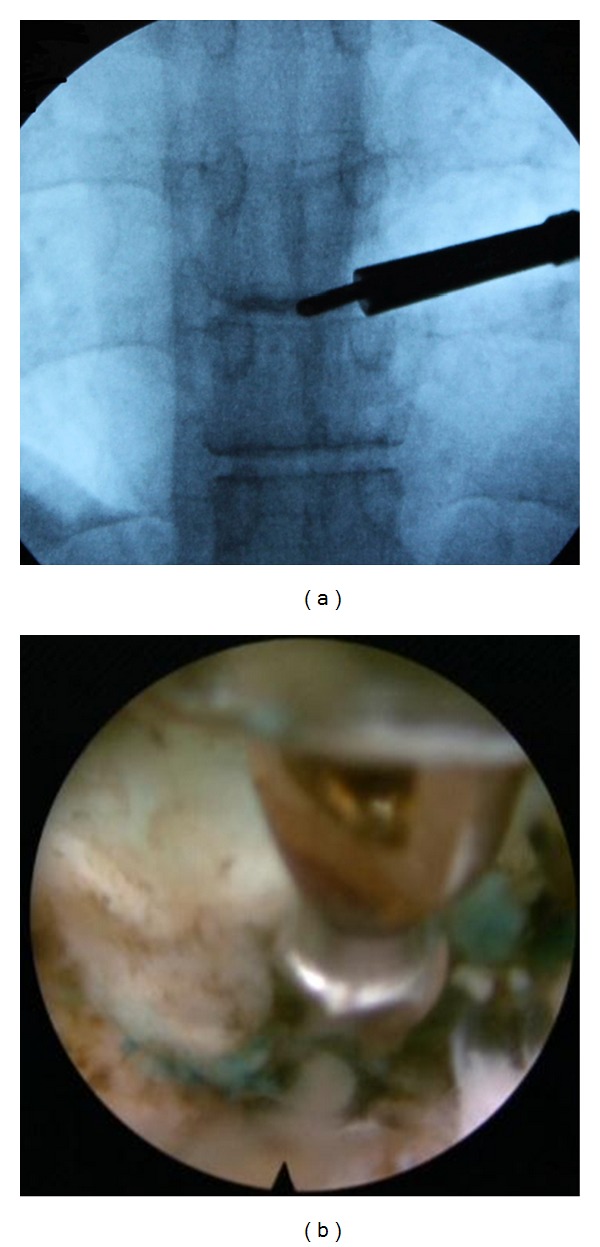
An Ellman radiofrequency probe (a) and a shaver (b) were used to expose the foraminal structure.

**Figure 5:**
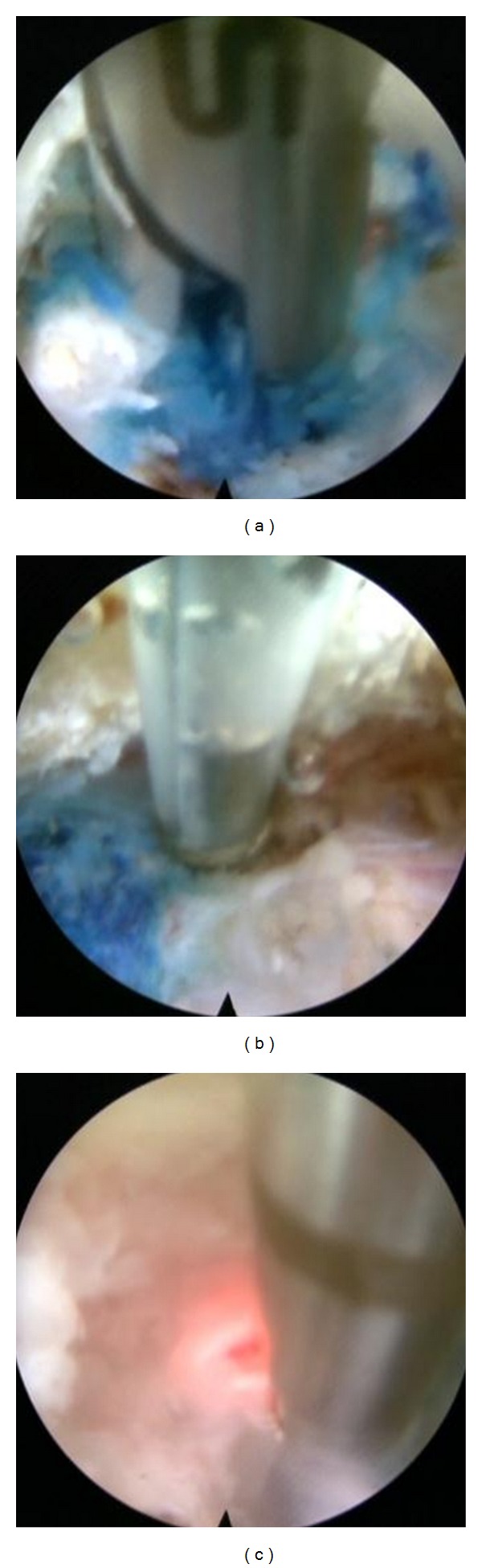
The herniated disc material was removed using a grasper (a), radiofrequency (b), and the Holmium-YAG laser (c).

To perform foraminotomy, we first titled the cannula to expose the foraminal epidural space. We then used an Ellman radiofrequency probe (Ellman International, New York, USA) and a shaver to expose the facet medially and rib head laterally (Figure 4). The radiofrequency, as well as the Holmium-YAG laser, was used to remove scar tissue, when needed. A burr, bone shaver (Richard Wolf Medical Instruments Corporation, Vernon Hills, IL, USA), and the Holmium-YAG laser were used to undercut the facet and rib head, when necessary, to enlarge the foramen so the working cannula could be easily advanced to the inner foraminal zone. Once adequate foraminotomy was achieved, the inferior pedicle, disc, epidural space, and exiting spinal nerve root were exposed. Herniated disc material was then removed using a grasper, radiofrequency, and the laser (Figure 5). At the end of the procedure, free movement of the thecal sac was visible. After satisfactory decompression had been achieved, the endoscope was removed, and the wound was covered with a sterile strip.

## 3. Results

The treated disc levels included T5-6 (1), T6-7 (3), T7-8 (4), T8-9 (2), T9-10 (2), and T12-L1 (2). One patient had herniation at T6-7 and T7-8. The chief complain of these patients was mid back pain with or without radiation (Table 1).

The patients (male: 7; female: 6; age: 40–69) were followed up for more than 6 months. At the final followup (mean: 17 months; range: 6–41 months), patient self-reported satisfactory rate (excellent and good results) was 76.9%. The mean VAS for mid back pain was improved from 9.1 to 4.2, and the mean ODI was improved from 61.0 to 43.8 (Table 1). The average operation time for each herniated disc was about 50 minutes. Blood loss during the surgery was minimal to none. Only one complication of postoperative spinal positional headache occurred and the patient was successfully treated with epidural blood patch. No other complications were observed or reported during or after the surgery. One patient had recurrent thoracic disc herniation 8 months after the initial surgery. None of the patients experienced worsening of symptoms. When asked if they would undergo the same procedure again if needed in the future, 12 of the 13 patients said yes.

**Figure 6:**
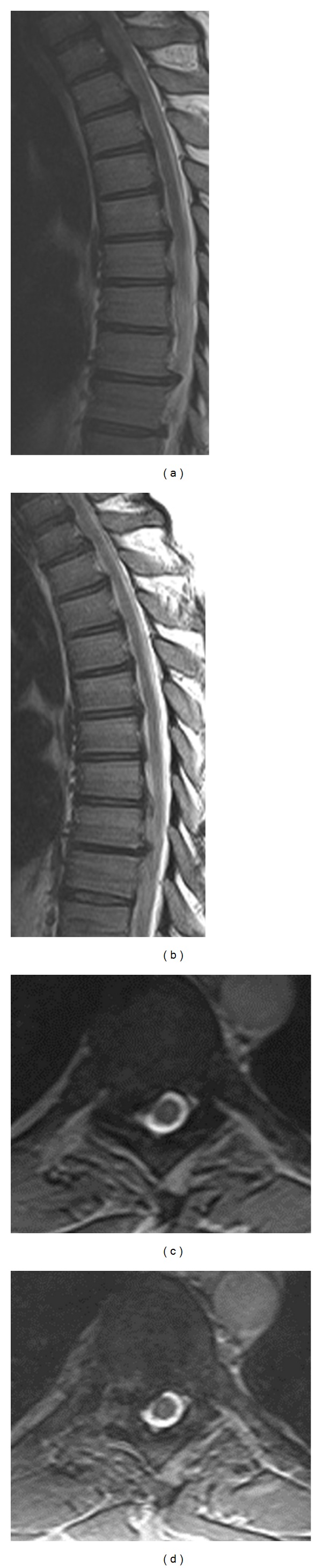
Preoperative MRI images of a T8-9 disc herniation compressing the spinal cord, which caused the patient to have mid back pain radiating to the shoulder blade ((a) and (b)). Postoperative MRI images showing removal of the extruded disc material ((c) and (d)).

Adequate decompression of the spinal cord was confirmed by postoperative MRI (Figure 6).

## 4. Discussion

Surgical treatment for thoracic herniation has evolved from the posterior approach to posterolateral and anterior approaches and from open surgery to minimally invasive surgery. To reduce access-induced complications and to improve surgical outcomes, various surgical techniques have been developed over the years. The literature review shows that minimally invasive techniques assisted with endoscopic or microscopic visualization have gained tremendous popularity in recent years. An analysis of a national database showed that utilizing minimally invasive techniques to treat thoracic disc herniation has become a new trend [29]. Despite the advancement in surgical instruments and techniques, surgically treating thoracic herniation remains a challenge because of the anatomical characteristics of the thoracic spine. Currently there are still no universally agreed upon indications for surgery, and the optimal type of decompression method is still controversial. Until a gold standard treatment is established, surgeons worldwide will employ different surgical techniques to treat thoracic disc herniations. And the choice of the technique will be dependent on the surgeon's training background, clinical experience, and personal preference.

Techniques using transforaminal approaches to treat thoracic disc herniation have a few advantages. The techniques generally need to remove only a small, lateral part of the facet joint to gain access for surgical and visualization instruments, and they generally do not require the resection of the unilateral facet joint and the caudal pedicle. Compared with posterior and anterior approaches, transforaminal approaches preserve postoperative spinal stability by avoiding resection of posterior vertebral elements and significantly reduce operative blood loss and postoperative pain by avoiding soft tissue dissection.

In our case series, thoracic disc herniations occurred at a wide range of disc levels (from T5-6 to T12-L1). Severe mid back pain with or without radiation was the chief complaint among all the patients treated. All patients reported immediate pain relief after the surgery, and at the final followup, the majority of the patients were still satisfied with the surgical outcome. This encouraging result suggests that our surgical technique is effective in improving the symptoms of thoracic herniations at different disc levels. When using a similar technique to treat soft thoracic disc herniations, Choi et al. also achieved satisfying results [28], which indicates the technique is reproducible.

In our study, at the final followup, 3 of the 13 patients (patients 1, 2, and 9 in Table 1) reported worsened functionality, as assessed by ODI scores. However, the worsened scores were most likely caused by factors unrelated to the original thoracic surgery. Before undergoing the thoracic discectomy at our center, patient number 1 had lumbar discectomy at L4-5 and L5-S1 levels. At the time when the patient answered the ODI questionnaire for our final followup assessment, the patient was suffering from recurrent L4-5 and L5-S1 herniations, which might be the reason that the patient gave poor ODI scores. Patient number 2 gave positive feedback right after the thoracic surgery, but she developed lumbar spondylolisthesis later. And at the time when the patient answered the ODI questionnaire, she was suffering from a broken ankle, which resulted in a loss of feeling in the foot. Patient number 9 had another herniation at T6-7, for which she was suggested to have another surgery. Despite the poor ODI scores at the final followup, when asked if they would consider the same surgery again if necessary in the future, all of the three patients said yes. This suggests that our technique is well accepted by the patients.

Compared with traditional surgical treatment, our endoscopic transforaminal technique offers a few advantages. Small incision and minimal bone removal reduce postoperative pain and ensure fast recovery. Local anesthesia enhances safety and further shortens the recovery. And excellent visualization provided by the endoscope ensures adequate decompression of the nerve. Moreover, same-day surgery with no need for hospital stay significantly reduces the total treatment cost. The low complication rate (0.08%, 1 of 13) and high patient self-reported satisfactory rate (76.9%) suggest that the technique is safe and effective in treating symptomatic soft thoracic disc herniation.

However, like all other surgical techniques, our technique also has limitations. First, the technique is not indicated for sequestrated thoracic disc herniation. And it is extremely challenging to remove large central herniations in patients with severe spinal stenosis. Patients with these conditions are generally referred to surgeons specializing in performing traditional open spine surgery, or thoracotomy. Because the thoracic spinal cord is highly susceptible to injury due to the anatomical nature of the thoracic spine, our technique requires the surgeon to have great surgical skills and considerable amount of experience with endoscopic surgery.

## 5. Conclusions

For carefully selected patients, endoscopic transforaminal thoracic discectomy and foraminotomy is a safe and effective treatment option for symptomatic soft thoracic disc herniation.

## Figures and Tables

**Table 1:** Patient baseline characteristics and clinical outcomes.

Case number	Age	Sex	Level	Main symptoms	Follow-up (M)	Pre-VAS	Post-VAS	Pre-ODI	Post-ODI	MacNab
1	59	F	T12-L1	Low back and mid back pain, leg pain	33.5	10	5	42	52	Good
2	43	F	T9-10	Mid back pain	32.5	10	7	56	88	Fair
3	40	M	T9-10 (R)/T9-10 (L)	Mid back pain	18	10	2	42	12	Excellent
4	56	F	T6-7	Mid back pain	13	7	4	60	52	Good
5	48	F	T6-7	Mid back pain	13	9	0	66	6	Excellent
6	52	M	T8-9	Mid back pain, upper back pain	13	10	5	92	62	Good
7	57	F	T5-6	Mid back pain, low back pain, and neck pain	12	10	9	70	58	Good
8	69	F	T7-8	Mid back pain, right chest pain	11.5	9	6	58	54	Poor
9	48	M	T7-8	Mid back pain, right chest pain radiates to abdomen	6.5	9	5	60	66	Excellent
10	32	M	T8-9	Mid back pain radiates to shoulder blade	6	6	4	36	18	Good
11	59	M	T6-7, T7-8	Mid back pain radiates to chest	15	10	6	62	54	Fair
12	54	M	T12-L1	Mid back pain	41	8	1	78	32	Excellent
13	51	M	T7-8	Mid back pain radiates to left side chest and rib	6	10	1	70	16	Excellent

VAS: visual analog scale, ODI: Oswestry Disability Index, Pre: preoperative, Post: postoperative.
